# Succesful Use of Lipid Emulsion Theraphy in a Case of Extremely High-Dose Olanzapine Intoxication

**DOI:** 10.5152/TJAR.2022.21315

**Published:** 2023-02-01

**Authors:** Meltem Şimşek, Fatma Yıldırım, Habibenur Şahin, Emine Banu Akay

**Affiliations:** 1 Department of Internal Medicine and Intensive Care, University of Health Sciences, Dışkapı Yıldırım Beyazıt Training and Research Hospital, Ankara, Turkey; 2 Department of Biochemistry, University of Health Sciences, Dışkapı Yıldırım Beyazıt Training and Research Hospital, Ankara, Turkey

**Keywords:** Intensive care, pharmacology, psychiatry, suicide, toxicology

## Abstract

In olanzapine intoxication, alterations in consciousness defined as “agitation despite sedation,” as well as cardiovascular and extrapyramidal side effects due to anticholinergic effects, can be seen. In this case report, we aimed to present a patient who took a very high dose of olanzapine for suicide purposes and later benefitted from intravenous lipid emulsion treatment. A 20-year-old male patient, who took 840 mg of olanzapine for suicide, was brought to the emergency room, and when the Glasgow Coma Scale was 5, he was intubated and given a single dose of activated charcoal. Later, he was admitted to the intensive care unit and was intubated. The olanzapine level was measured as 653 μg L^−1^. The patient was started on lipid emulsion treatment and woke up in the sixth hour. In addition to the lack of strong evidence regarding the use of lipid emulsion treatment in olanzapine intoxication, it is seen that lipid therapy has been used successfully in patients. Compared with the cases in the literature, lipid emulsion treatment was successfully applied in our case, where the blood olanzapine level was very high. Although there is no evidence-based treatment for olanzapine intoxication, we believe that lipid emulsion treatment has a positive effect on neurological recovery and survival.

**Main Points**

- Intoxications of antipsychotic drugs used in the treatment of schizophrenia are quite common.
- The main treatment of Olanzapine intoxication, which is one of the atypical antipsychotic drugs, consists of treatments supportive.
- Successful results of lipid emulsion therapy take their place in the literature when no response to supportive treatment can be obtained, In cases of severe intoxication.

## Introduction

Atypical antipsychotic drugs (AAD) are frequently used in the treatment of schizophrenia, replacing first-generation antipsychotics due to their minimal extrapyramidal side effects (EPSE) (acute dystonia, Parkinsonism, ataxia, and tardive dyskinesia). Atypical agents cause side effects such as neurocognitive impairment and negative symptoms more frequently by blocking the mesolimbic D2 receptors (nigrostriatal and prefrontal cortex) selectively.^1^ Toxic exposure associated with atypical agents is seen as an ongoing problem in the United States and many regions.^2,3^ Although it causes mild to moderate toxicity in most of the patients and does not show symptoms in acute intoxication caused by AAD, toxic and lethal doses are very variable. Toxic effects begin in 1-2 hours and peak in 4-6 hours. In addition, despite the fact recovery usually begins in 12-48 hours, cases extending up to 6 days have been reported.^1^

Olanzapine, one of the AAD, antagonizes alpha-1, histamine-1, and muscarinic-1 receptors. At toxic doses, alpha-1 receptor blockage causes dizziness, orthostatic hypotension, reflex tachycardia, miosis, nasal decongestion, histamine-1 receptor blockage, central nervous system depression, apathy, and hypotension. Besides, muscarinic-1 receptor blockage causes agitation, hallucinations, memory disorders, dry skin and mucous membranes, hypertension, constipation, mydriasis, blurred vision, tachycardia, and urinary retention. Extrapyramidal side effects and neuroleptic malignant syndrome are rare. In many case reports, fluctuations in the state of consciousness stated as “agitation despite sedation” have been described in olanzapine overdose.^4^ In electrocardiography, it may cause QT and QRS enlargement, as well as hyperglycaemia, an increase in liver enzymes, agranulocytosis, leukopenia, and neutropenia.

Although, the main treatment of AAD toxication is supportive treatment in addition protection of the airway, providing respiration, and maintaining circulation and which are the general approach to intoxication are the main treatment. In the treatment of hypotension secondary to alpha-adrenergic block, isotonic crystalloids are preferred first, while noradrenaline (NA) and phenylephrine are given in resistant hypotension. Besides, QT prolongation is treated using type 1A antiarrhythmic (quinidine, procainamide), type 1C antiarrhythmic (flecainide, propranolol), and type III antiarrhythmic(amiodarone, sotalol, ibutilide), and QRS prolongation is treated with sodium ­bicarbonate. Benzodiazepines are the first-choice drugs in seizures due to poisoning. A single dose of activated charcoal is recommended. Physostigmine, a short-acting anticholinesterase inhibitor that successfully reverses the effects of the olanzapine-induced anticholinergic syndrome, is also a recommended treatment. In the case of refractory toxicity, it is stated that lipid emulsion therapy can be used in severe poisoning if standard treatment cannot be improved.^4^

In this case report, we aimed to present a 20-year-old male patient, who was intubated and followed up in the intensive care unit, who took a very high dose of olanzapine for suicide purposes, showed rapid recovery after intravenous lipid emulsion treatment (LET), and was transferred to the service after leaving the invasive mechanical ventilator (IMV).

## Case Presentation

A 20-year-old male with known mental retardation and attention-deficit hyperactivity disorder is brought to the emergency room because he is unconscious at home. We learned from the history of the patient that he took 3 boxes of olanzapine (84 tablets from a 10 mg tablet) for suicide. The patient, who also had a suicide attempt that required a 1-week hospitalization 3 years ago, was intubated because of the Glasgow Coma Scale (GCS) of 5 when he was brought to the emergency room. The patient, who was given a single dose of activated charcoal in the emergency department, was admitted to the intensive care unit (ICU) under IMV support (Tables 1 and 2).

During the ICU follow-up of the patient, his body temperature increased to 39.5°C. During the follow-up, the heart rate ranged between 110 and 160 per minute. Despite not receiving sedation, the patient did not have spontaneous breathing and alertness. Nitrate infusion therapy was initiated in the patient whose blood pressure was measured as arterial 180/110. During the follow-up of the patient, liver function tests, kidney function tests, CK, CK-MB, and LDH values were not found to be abnormal. QT and QRS follow-up was performed on the patient and no prolongation was found. The level of olanzapine (serum/plasma) sent from the patient was measured as 653 µg L^−1^ (normal: 20-80). The patient was started on LET (ClinOleic^®^, a lipid emulsion composed of 80% olive oil and 20% soybean oil) as 1.5 mL^−1^ kg^−1^ half an hour and 1.5 mL min^−1^ infusion. A total of 1000 mL of lipid solution was given to the patient. The patient woke up in the sixth hour after the administration of LET. The patient, who had complete cooperation and orientation, was extubated because his haemodynamics were also stable. The patient was transferred to the ward.

## Discussion

In this study, we presented the successful use of LET in our patient, who was taking olanzapine for suicide purposes, whose blood olanzapine level was very high, and who needed IMV.

Considering the literature, it is possible to say that evidence-based information is limited in olanzapine intoxication. In addition to supportive therapy, a single dose of activated charcoal is recommended to prevent and reduce gastrointestinal absorption and physostigmine, a short-acting anticholinesterase inhibitor, to relieve symptoms related to anticholinergic side effects.^4^ It is thought that olanzapine, a lipophilic drug, can be removed from the body via the kidney as transport from cells to plasma with LET.^5^ As a second mechanism, long-chain fatty acids are thought to increase contractility in cardiac myocyte cells by affecting voltage-dependent calcium channels and sodium channels, and this mechanism has also been suggested as responsible for their haemodynamic positive effects.^6^ The general indications for LET in the literature are local anaesthetics, tricyclic antidepressants, flecainide, verapamil, diltiazem, bupropion, lamotrigine, beta-blockers, quetiapine, haloperidol, and lipophilic drug intoxications such as cocaine. Although there are no clinical studies, there are many case reports. When the literature was reviewed, LET was given to females aged 35 and 64 years, in addition to the standard treatment, the need for vasopressors, invasive mechanical ventilation, and haemodynamic instability due to quetiapine intoxication. Both patients were extubated and the vasopressor was discontinued.^7^ In a study consisting of 40 cases of clozapine intoxication divided into 2 equal groups, the control group was given only the standard treatment, while the other group was given LET in addition to the standard treatment. Glasgow Coma Scale was found significantly higher and prolonged QTc was found lower at the 6th and 12th hours of admission. In addition, hospitalization rate in the group given LET was found lower. Furthermore, there were no adverse effects related to its administration.^8^ It is thought that the side effects of LET may cause more serious consequences than olanzapine intoxication. Lipid emulsion treatment may cause side effects such as hyperlipidaemia, seizures, allergic reactions, fat embolism, jaundice, and pulmonary oedema. However, it should be noted that these effects are very rare.^6^ No side effects were encountered in our case.

In the case presented by Yeniocak et al^9^, the blood olanzapine level of the patient who took 250 mg olanzapine at the age of 30 was measured as 257.8 ng mL^−1^ (normal: 20-80) and a 20% LET was applied. The patient, who came with agitation and confusion, showed neurological improvement in the third hour, and the blood olanzapine level was measured as 74 ng mL^−1^ at the end of the fifth hour. As far as we know, in the case report presented by Lennestål et al^10^ which had the ­highest olanzapine level in the literature (815 µg L^−1^), olanzapine half-life was evaluated as 2 phases, while the half-life of the first 3 days was 22 hours, and the second phase half-life was 2-3 days as stated. With this study, it was concluded that olanzapine level over 100 µg L^−1^ is potentially toxic. Olanzapine level reaches its peak plasma level 3-6 hours after ingestion. In our case, the blood level could not be measured on the third day due to technical limitations. It has been reported that the serum olanzapine level in fatal cases varies from 237 to 5200 µg L^−1^.^11,12^ In our case, the blood olanzapine level was measured as 653 µg L^−1^ and it can be considered to be very high according to the literature.

In our case, although the blood olanzapine level was higher than the case presentation by Yeniocak et al^9^, it was neurologically improved at the sixth hour with 1000 mL lipid treatment (2700 mL LET was given in the case of Yeniocak et al^9^). Yurtlu et al^13^ stated that the GCS of a 39-year-old female increased from 11 to 15 with 100 mL of 20% lipid emulsion in 15 minutes. Considering these cases presented in the literature, there is no common information about how long or how much LET should be given.

## Conclusion

Although there is no evidence-based treatment for olanzapine intoxication, we believe that LET has a positive effect on neurological recovery and survival.

## Figures and Tables

**Table 1.** Vital Signs

Body temperature, °C	36.3
Heart rate, beats min^−1^	118
Tension arterial, mmHg	160/110
Number of breaths, breaths min^−1^	22
O_2_ saturation, %	90

**Table 2.** Laboratory Findings at the Time of Admission

Laboratory Values	Result (Normal Values)
pH	7.36 (7.35-7.45)
pCO_2_, mmHg	41.9 (35-48)
pO_2_, mmHg	67.4 (82-108)
HCO3, mmol L^−1^	23.2 (21-26)
Lactate, mmol L^−1^	1.0 (0.5-1.6)
Glucose, mg dL^−1^	104 (70-100)
Urea, mg dL^−1^	28 (16-38)
Creatinine, mg dL^−1^	0.65 (0.5-0.9)
Total bilirubin, mg dL^−1^	0.44 (0-1.2)
Direct bilirubin, mg dL^−1^	0.1 (0-0.3)
AST, U L^−1^	29 (0-32)
ALT, U L^−1^	15 (0-33)
Calcium, mg dL^−1^	9.2 (8.6-10.2)
Creatine kinase (CK), U L^−1^	25 (0-170)
CK-MB, U L^−1^	49 (0-25)
Lactate dehydrogenase, U L^−1^	212 (135-225)
Sodium, mEq L^−1^	139 (136-145)
Potassium, mEq L^−1^	4.09 (0.5-5.1)
Chlorine, mEq L^−1^	107 (98-107)
White blood cell count, ×10³ µL^−1^	5.5 (3.57-11.01)
Haemoglobin, g dL^−1^	12.7 (11.7-15.5)
Platelet, ×10³ µL^−1^	233 (105-372)
Prothrombin time, seconds	12.8 (8.4-10.6)
INR	1.1 (0.8-1.22)
Activated partial thromboplastin time, seconds	31.3 (23.9-33.2)
d-Dimer, µg mL^−1^	0.28 (0-0.5)
Fibrinogen, mg dL^−1^	321 (193-412)
C-reactive protein, mg L^−1^	2.39 (<5)
Procalcitonin, µg L^−1^	0.05 (<0.05)
Troponin, ng mL^−1^	<0.1 (0-0.3)

AST, aspartate aminotransferase; ALT, Alanin aminotransferase; CK-MB, Creatine kinase myocardial band; INR, International normalized ratio.
